# Grid-based stochastic search for hierarchical gene-gene interactions in population-based genetic studies of common human diseases

**DOI:** 10.1186/s13040-017-0139-3

**Published:** 2017-05-30

**Authors:** Jason H. Moore, Peter C. Andrews, Randal S. Olson, Sarah E. Carlson, Curt R. Larock, Mario J. Bulhoes, James P. O’Connor, Ellen M. Greytak, Steven L. Armentrout

**Affiliations:** 1 Department of Biostatistics and Epidemiology, Institute for Biomedical Informatics, Perelman School of Medicine, University of Pennsylvania, Philadelphia, 19104 PA USA; 2 Parabon Computation, Inc, Reston, 20190 VA USA; 3 Parabon NanoLabs, Inc, Reston, 20190 VA USA

**Keywords:** Bioinformatics, Epistasis, Genome-wide Association study, Machine learning, Common diseases

## Abstract

**Background:**

Large-scale genetic studies of common human diseases have focused almost exclusively on the independent main effects of single-nucleotide polymorphisms (SNPs) on disease susceptibility. These studies have had some success, but much of the genetic architecture of common disease remains unexplained. Attention is now turning to detecting SNPs that impact disease susceptibility in the context of other genetic factors and environmental exposures. These context-dependent genetic effects can manifest themselves as non-additive interactions, which are more challenging to model using parametric statistical approaches. The dimensionality that results from a multitude of genotype combinations, which results from considering many SNPs simultaneously, renders these approaches underpowered. We previously developed the multifactor dimensionality reduction (MDR) approach as a nonparametric and genetic model-free machine learning alternative. Approaches such as MDR can improve the power to detect gene-gene interactions but are limited in their ability to exhaustively consider SNP combinations in genome-wide association studies (GWAS), due to the combinatorial explosion of the search space. We introduce here a stochastic search algorithm called Crush for the application of MDR to modeling high-order gene-gene interactions in genome-wide data. The Crush-MDR approach uses expert knowledge to guide probabilistic searches within a framework that capitalizes on the use of biological knowledge to filter gene sets prior to analysis. Here we evaluated the ability of Crush-MDR to detect hierarchical sets of interacting SNPs using a biology-based simulation strategy that assumes non-additive interactions within genes and additivity in genetic effects between sets of genes within a biochemical pathway.

**Results:**

We show that Crush-MDR is able to identify genetic effects at the gene or pathway level significantly better than a baseline random search with the same number of model evaluations. We then applied the same methodology to a GWAS for Alzheimer’s disease and showed base level validation that Crush-MDR was able to identify a set of interacting genes with biological ties to Alzheimer’s disease.

**Conclusions:**

We discuss the role of stochastic search and cloud computing for detecting complex genetic effects in genome-wide data.

## Introduction

Genomics has provided a better understanding of how DNA sequence variations influence disease susceptibility through biomolecular interactions, including protein-DNA, protein-RNA, RNA-RNA, and protein-protein binding within the context of a gene regulatory region. For example, Cowper-Sal. Lari et al. showed that single-nucleotide polymorphisms (SNPs) associated with breast cancer in genome-wide association studies (GWAS) are enriched in FOXA1 transcription factor binding sites resulting in allele-specific gene expression in cancer cells [1]. These same kinds of biomolecular interactions drive biochemical pathways and physiological systems, propagating genetic effects to the disease phenotype level. Given the complexity of biomolecular interactions that connect DNA sequences to anatomical and physiological perturbations, ultimately driving phenotypes from the healthy to diseased range, it is logical to assume that some genetic risk factors will manifest themselves as non-additive gene-gene interactions at the population level [2]. Revealing the complexity of the genetic architecture of common human diseases such as Alzheimer’s disease or essential hypertension will require a combination of computational, mathematical, and statistical methods that embrace, rather than ignore, the hierarchical and interactive nature of the genetic signals that are propagated to a healthy or disease phenotype [3, 4].

Bateson, who recognized that one gene could modify the effects of another gene thereby skewing Mendelian expectations, was the first to describe this concept of gene-gene interaction, or epistasis [5]. This was a biological concept of gene action in cells [6]. In contrast, Fisher described epistasis as deviation from additivity in a linear statistical model such as analysis of variance [7]. This population-level concept is based on a statistical summary of many individuals. The relationship between biological and statistical epistasis is an unsolved problem in human genetics, [8, 9] but it is critical if we are to use population-based measures of genetic association to guide the development of new treatments that target biological processes at the cellular level. We focus here on the detection of epistasis in human populations using computational methods designed to detect non-additive gene-gene interactions in high-dimensional data.

Parametric statistical methods, such as logistic regression, are commonly employed to detect genetic associations. These approaches have nice mathematical properties and produce parameter estimates that can be interpreted as measures of risk. However, there are several disadvantages, including the assumption imposed by a specific mathematical model (i.e. a linear regression function with a logit link) and reduced power to detect interactions. Machine learning provides a nonparametric and genetic model-free alternative by harnessing the power of adaptive algorithms, which are designed to detect complex patterns in high-dimensional data [10]. A key difference is that machine learning allows the data to determine the model rather than forcing the data to fit a preconceived notion of what a model should look like. Several recent reviews highlight the need for new methods [11] and discuss and compare different strategies for detecting statistical epistasis [3, 4, 12]. The methods reviewed by Cordell [3] include novel approaches such as combinatorial partitioning, [13, 14] multifactor dimensionality reduction (MDR), [15, 16] and logic regression [17, 18]. We focus here on the MDR method.

MDR was developed as a nonparametric (i.e. no parameters are estimated) and genetic model-free (i.e. no genetic model is assumed) data mining and machine learning strategy for identifying combinations of genetic and environmental factors that are predictive of a discrete clinical endpoint [15, 16, 19–22]. Unlike most other methods, MDR was designed to detect interactions in the absence of detectable marginal effects and thus complements statistical approaches such as logistic regression and machine learning methods such as random forests and neural networks.

At the heart of the MDR approach is a feature or attribute construction algorithm that creates a new variable or attribute by pooling genotypes from multiple SNPs [21]. The general process of defining a new attribute as a function of two or more other attributes is referred to as constructive induction, or attribute construction, and was first described by Michalski [23]. Constructive induction, using the MDR kernel, is accomplished as follows. Given a threshold *T*, a multilocus genotype combination is considered high-risk if the ratio of cases (subjects with disease) to controls (healthy subjects) exceeds *T*, otherwise it is considered low-risk. Genotype combinations considered to be high-risk are labeled *G*_*1*_ while those considered low-risk are labeled *G*_*0*_. This process constructs a new one-dimensional attribute with values of *G*_*0*_ and *G*_*1*_. It is this new single variable that is assessed, using any classification method. The MDR method is based on the idea that changing the representation space of the data will make it easier for methods such as logistic regression, classification trees, or a naive Bayes classifier to detect attribute dependencies. As such, MDR significantly complements other classification methods such as those reviewed by Hastie et al. [24]. This method has been evaluated in numerous simulation studies, [19, 25] and a user-friendly open-source MDR software package written in Java is freely available [22, 26].

The MDR method was first implemented as a combinatorial approach that performed an exhaustive search across all two-way, three-way, and higher-order combinations of genetic variants for a best model. As reviewed by Moore et al., [4] this is not practical in the context of GWAS due to the exponential explosion of the search space. High-performance computing can only enable exploration of all two-way and perhaps three-way combinations of genetic variants in a typical genome-wide scan [27, 28]. Moore et al. outline two different approaches to MDR analysis in genome-wide data [4]. The first is to use computational filter methods, such as ReliefF, to reduce the number of variants to those most likely to be involved in gene-gene interactions [29, 30]. Another approach is to use biological filters to limit the analysis to variants in genes within the same pathway or those that exhibit protein-protein interactions [31]. A third approach, which we explore here, is to use stochastic search algorithms that probabilistically pick genetic variants for consideration by MDR.

We introduce here a multiobjective evolutionary search engine for MDR implemented in the Parabon Crush^™^ statistical analysis application. Crush employs an opportunistic evolutionary strategy designed to maximally utilize an arbitrary collection of distributed compute nodes [32]. MDR’s evaluation of candidate groups of SNPs serves as one of the critical objectives in the Crush-MDR application, the details of which are provided below. The methods introduced here will facilitate the search for gene-gene interactions on a genome-wide scale using cloud computing resources.

## Methods

There are five major components to the Crush-MDR method. The first is the MDR machine learning component. The second is the Crush stochastic search framework. The third is the expert knowledge component. The fourth is the multiobjective optimization component. The final component is the Software as a Service (SaaS) framework, which combines all of these components for cloud-based analysis. We describe each of these in turn and then describe methods for evaluating Crush-MDR using simulated data.

### Modeling gene-gene interactions using MDR

The goal of this component is to provide a machine learning framework for modeling non-additive gene-gene interactions. As described above, MDR uses constructive induction to collapse multiple genetic variants to a single new feature that is used to classify cases and controls. We specifically used the quantitative MDR or QMDR extension to enable the modeling of quantitative traits [33, 34]. Here, QMDR identifies those genotype combinations with mean trait levels above or below or the global mean and then constructs a new feature by collapsing all the genotypes above the mean into one group and all those below the mean into another group. The genotype groups are then compared using a *t*-test. As presented in detail by Gui et al., [33] constructive induction by QMDR is performed as follows:

1. Assume there are m SNPs in the dataset; to examine a K-order interaction, select K SNPs from the m total SNPs.
2. For each multi-locus genotype combination defined by the K SNPs, calculate the mean value and compare it with the overall mean.
3. If the mean value from the genotype combination is larger than the overall mean, the corresponding genotype is considered high-level. Otherwise, it is considered low-level. Once all of the genotypes are labeled ‘high-level’ and ‘low-level’, a new binary attribute is created by pooling the “high-level” genotype combinations into one group and the “low-level” into another group.

Once a new attribute is constructed, high and low level groups defined by QMDR are compared using a *t*-test. The t-statistic is used as a training score to choose a best model. The cross-validation procedure for QMDR is the same as that used in the discrete version of MDR. The difference is that the training score and testing score are defined from the *t*-test (replacing training and testing accuracy). The training score is used to determine the best K-order interaction model, and the maximum testing score is used to identify the best overall model. It is important to note that the discrete version of MDR can be used here when the endpoint is binary.

### Stochastic search for gene-gene interactions using Crush

The goal of this component is to provide a stochastic search algorithm for MDR to enable the search for gene-gene interactions in genome-wide data without the computational burden of exhaustively evaluating all combinations. To achieve this goal, we implemented MDR as an objective function under Parabon Crush, which performs stochastic search using an opportunistic evolution search algorithm over candidate solutions comprised of one or more SNPs [32]. Crush’s opportunistic evolution algorithm is designed to maximize the efficiency and throughput of evolutionary search across intermittently available compute nodes, such as Amazon’s EC2 Spot Instance. Briefly stated, Crush uses a central steady-state evolutionary process, controlled by the client (launching) application, to populate and remotely execute independent evolutionary tasks on participating compute nodes. Crush-MDR employs generational evolution on compute nodes, which periodically return subpopulations of candidate solutions for incorporation into the global steady-state population. This process of launching evolutionary tasks from the client, evolving candidate solutions on distributed compute nodes, and incorporating resulting subpopulations into the master steady-state population on the client, is repeated until a termination condition is reached. We describe below the computational details of how good MDR models in a population of many models are selected and then varied through mutation and crossover. Key to this process is tournament selection that randomly picks three MDR models from the population of all models and then picks the best one according to the score for that model and the complexity as measured by the number of SNPs. The approach to balancing these two criteria is called Pareto optimization and is described further below in the evaluation section. Together these form the nondominated sorting genetic algorithm (NSGA II) method that is described in detail below [35].

Crush-MDR begins by generating a population of MDR models. The algorithm first chooses the number of SNPs (K) for a model in the initial population by randomly selecting a value between one and five. This is based on a configurable value of how many SNPs should be in a model, plus or minus a delta. The default settings are three SNPs per model, plus or minus two giving a range of one to five. The SNPs for each model are selected 50% of the time at random with equal probability and 50% of the time using expert knowledge (see below). This initial set of models is then evaluated (see below). Breeding of candidate solutions in the global steady-state population is performed by any-point crossover of two parent solutions picked by two tournament selections from the population of evaluated solutions. The first tournament selects the solution with the lowest Pareto rank (see discussion of Pareto optimization below) and best (highest) crowding distance as computed by NSGA II [35]. The second tournament performs the same selection (lowest Pareto rank and best crowding distance) and then mutates the selected solution with a variable probability of 0.5 or 5% that an arbitrary SNP will be mutated for datasets with 1000 or 100 SNPs, respectively. The size of both tournaments is set to three. Any-point crossover is then performed on the solutions produced by the tournaments, and the resultant solutions are used as part of the initial population of a new evolutionary task. It is important to note that models with more than five SNPs can be generated through crossover.

Breeding of candidate solutions in the evolutionary task generational population is performed by crossover or mutation. Any-point crossover of two parent solutions picked by two tournament selections from the population of evaluated solutions is used 90% of the time. The first tournament of size six selects individuals with the lowest model order, while the second tournament of size three selects the solution with the lowest Pareto rank and best (highest) crowding distance as computed by the nondominated sorting genetic algorithm II (NSGA II). This tournament combination applies parsimony pressure to candidate solutions by favoring those with lower model order for the first parent solution. This is unlike other parsimony strategies, such as weighting factors or cutoff limits on model order, as it requires no *a priori* knowledge of the fitness space. The remaining 10% of the time solutions are randomly selected from the population and then mutated with a probability of 0.5% that an arbitrary SNP will be mutated.

### Expert-knowledge guided Crush-MDR

As discussed by Moore et al., expert knowledge is critical for identifying gene-gene interactions in genome-wide data [4]. Expert knowledge can come from any source that the investigator thinks might be important for guiding an algorithm to a more fruitful part of what is, practically speaking, an infinite search space. Since we are interested in identifying gene-gene interactions, we selected interaction information as an entropy-based measure of non-additive interactions [21]. A detailed description of this method and its implementation can be found in Moore and Hu, [36] and example applications can be found in Hu et al. and De et al. [34, 37]. Briefly, we measure the gain in information from phenotypic values due to the joint effects of each pair of SNPs above and beyond that provided by the independent main effects. A positive information gain is indicative of a synergistic interaction, while a negative score is indicative of correlation or redundancy (e.g. due to linkage disequilibrium), resulting in loss of information. We computed the information gain due to interaction for each pair of SNPs and then stored this expert knowledge about pairwise gene-gene interactions in a lookup table, which is used by Crush-MDR to construct models in the initial population. The lookup table approach was used by Pattin et al. for using protein-protein interactions as expert knowledge [31]. This sensible initialization approach seeds Crush-MDR models at the start of the algorithms with SNPs likely to be involved in interactions. The algorithm first picks the number of features in the initial models using a uniform distribution between one and five. The algorithm then probabilistically selects the features for that model from the list of all genetic variants. Each feature has a probability of 0.5 for being selected randomly with equal probability or from a lookup table based on expert knowledge indicating what features interact with the previously selected feature. The pairs of features selected using expert knowledge represent initial building blocks for the algorithm to work with. We have previously shown this type of sensible initialization is beneficial for these kinds of problems [30].

It is important to note that the entropy-based measures of interaction that we used as expert knowledge are not statistically independent of some of the interactions that are later modeled by MDR. This is in contrast to the example of Pattin et al. that used protein-protein interactions as expert knowledge that were statistically independent of the data being analyzed [31]. An advantage of Crush-MDR is that it can build models of any arbitrary size allowing it to potentially capture higher-order interactions. We selected this particular source of expert knowledge to provide Crush-MDR with some lower-order interactions that might be useful for higher-order models thus reducing the search space.

### Evaluating Crush-MDR models using multiple criteria

A common approach for addressing overfitting in data mining and machine learning is to use cross-validation as an estimate of the generalizability of a model. Unfortunately, implementation of cross-validation methods in conjunction with stochastic methods, such as evolutionary computation, can be complex, given that these algorithms are likely to find different models in each division of the data. Pareto optimization (reviewed in [38]) offers a viable alternative and has been shown to be effective in the context of evolutionary methods [39]. Pareto optimization balances several different model objectives that are each treated equally. We previously used classification accuracy and model size as our two objectives [40]. We have also used a third objective defined by the average interaction information of the SNPs in the model [41]. This extra objective rewards models for stronger gene-gene interactions. For a given population, models for which there are no better models as measured by accuracy, model size and interaction information are selected. This subset of Pareto-optimal models is referred to as the Pareto front. As described by Moore et al., a benefit of this approach is that it allows Crush-MDR to explore models that score well on the interaction scale but that might not have strong associations [41]. These models are selected, changed, and passed on to each new generation model in the evolutionary algorithm. Here we used the QMDR t-statistic as the measure of association. The algorithm works to jointly maximize the t-statistic and maximize the interaction information, while minimizing the model complexity. Putting pressure on the algorithm to explore smaller models is beneficial because these are the models that are more likely to generalize to independent data. At the same time, Pareto optimization also allows bigger models to be explored thus preserving the diversity of the SNPs represented to allow new models to be more effectively explored throughout the evolutionary process. This helps prevent the algorithm from becoming stalled or fixated on a local minima.

### Cloud-computing implementation of Crush-MDR

We implemented the Crush-MDR method as both stand-alone software and a Software as a Service (SaaS) application [42]. The SaaS implementation of Crush-MDR provides users with simple on-demand access to the Crush-MDR statistical algorithms through a standard web browser. In addition to these analytical capabilities, the SaaS interface includes capabilities for visualizing results (e.g., visualization of the Pareto front and visualization of statistical epistasis networks) and support for user collaboration and data sharing. Both the stand-alone and SaaS versions of Crush-MDR run on a parallel cloud computing infrastructure, [43] enabling massively parallel execution of evolutionary searches across a wide variety of computational resources, including public and private clouds, high performance computing clusters, and enterprise computing resources.

### Evaluation of Crush-MDR using simulation

We previously developed several approaches to simulating genetic association data with complex relationships between genotype and phenotype. Our Genetic Architecture Model Emulator for Testing and Evaluating Software (GAMETES) method uses penetrance functions to probabilistically specify risk of disease given different combinations of genotypes from two or more SNPs [44, 45]. We developed here an extension of GAMETES that allows multiple sets of interacting SNPs to be combined additively to produce hierarchical models that simulate the action of two or more genes or biochemical pathways. We also extended GAMETES to simulate quantitative traits that are more common now that genetic data is being integrated with proteomics data, metabolomics data, and clinical data from electronic health records. These extensions allowed for more complex and higher-order models to be easily generated for the evaluation of Crush-MDR.

Using the GAMETES extension method described above, we designed a comprehensive simulation study to evaluate Crush-MDR across different genetic models exhibiting non-additive interactions, different effect sizes, and different data characteristics such as sample size. The biological framework used in the simulation is based on additive sets of non-additive gene-gene interactions within a biochemical pathway. Here, each gene produces a continuous protein product that is dependent on a non-additive interaction between the genotypes at two SNPs. This could be thought of as the interaction between two regulatory SNPs governing gene expression. We then combined the protein products from two or four genes additively to produce a final quantitative trait that is used as the phenotype in the Crush-MDR analysis.

The simulated data varied by number of functional genes (two and four), functional SNP allele frequencies (0.2 and 0.4), sample sizes (2000 and 8000), effect sizes measured in terms of heritability (0.001, 0.01, 0.1, and 0.2), and effect sizes due to standard deviation of quantitative traits (0.05, 0.1, 0.2, and 0.3). The chosen heritability values used represent broad-sense heritability for each pair of interacting SNPs within each gene. The standard deviation represents the dispersion of the quantitative trait around a mean value equal to the penetrance for that genotype combination. Thus, each pair of SNPs within a gene generates a quantitative trait (e.g. protein level) that is then summed across genes to generate the quantitative trait used as the phenotype in the Crush-MDR analysis. The goal of the Crush-MDR analysis was to correctly identify the four or eight functional SNPs from this hierarchical model consisting of additive units of non-additive gene-gene interactions. These SNPs were then added to 996 or 992 randomly generated SNPs representing the tag-SNPs from a single biochemical pathway embedded in a GWAS for a total of 1000 SNPs. A total of ten data sets were simulated for each combination of parameter settings. The results are presented as the success rate defined by the proportion of times the correct SNPs were identified across all 10 datasets.

### Application of Crush-MDR to the genetic analysis of Alzheimer’s disease

To evaluate performance on real data, Crush-MDR was applied to whole-genome sequence data from the Alzheimer’s Disease Neuroimaging Initiative (ADNI) [46]. Sequenced variants were filtered to biallelic SNPs with minor allele frequency > 0.05 and pairwise linkage disequilibrium < 0.8 using PLINK, [47] for a total of 1,321,689 ADNI SNPs. We constructed a list of 734 genes in the Alzheimer’s disease pathway using Ingenuity Pathway Analysis (IPA) and used Biofilter [48] to estimate pairwise biological interactions among this set of genes. A minimum Biofilter interaction threshold of four to six was chosen to yield a SNP set comparable in size to the simulated datasets (758 SNPs in 28 genes). For each subject, the hippocampal volume (HV) at baseline was calculated and normalized by intracranial volume, and this continuous variable was used as the phenotype for Crush-MDR analysis on 718 subjects. Initially, all pairs of factors were evaluated for pairwise entropy, and the maximum entropy and best partner for each SNP was recorded. A Crush-MDR evolutionary run was then performed using multiobjective optimization with three objectives: maximized MDR t-Statistic, maximized average entropy, and minimized model size.

## Results

### Evaluation of Crush-MDR using simulation

**Fig. 1:**
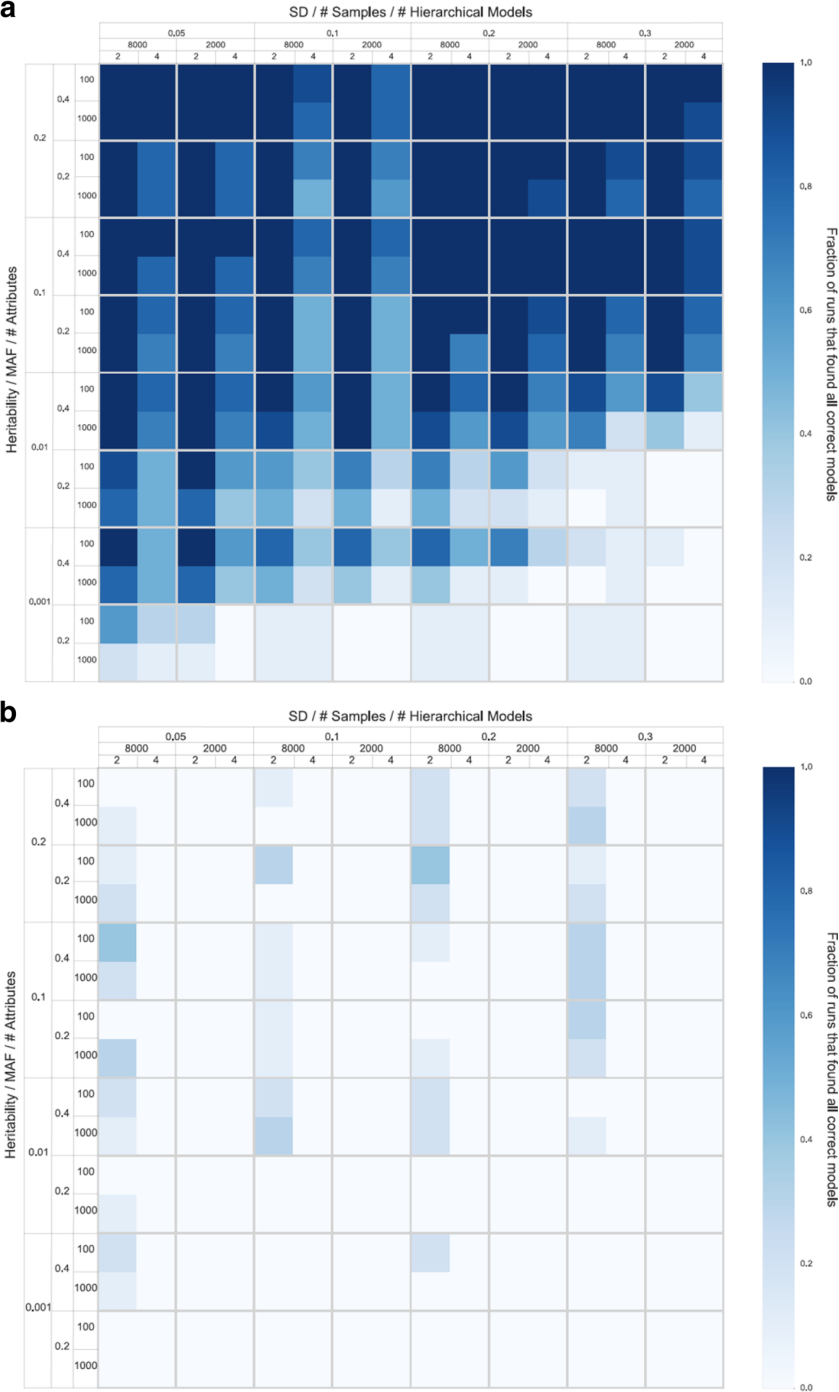
Heatmaps summarizing the results of the simulation study. The *dark blue* shading indicates a detection success rate of 80% or greater for each particular heritability, minor allele frequency (MAF; 0.2 and 0,4), total number of SNPs (100 and 1000), size of the embedded ‘target’ models (i.e. the number of SNPs they contain; 2 and 4), sample size (2000 and 8000), and trait standard deviation combination (0.05, 0.1, 0.2, and 0.3). Panel **a** represents the described Crush-MDR search. Panel **b** represents a random search with the same number of evaluations

The results of the simulation study are summarized as a heatmap in Fig. 1a. The dark blue shading indicates a detection success rate of 80% or greater for each particular heritability, minor allele frequency (MAF), total number of SNPs (100 and 1000), size of the embedded ‘target’ models (i.e. the number of SNPs they contain), sample size, and trait standard deviation combination. On average, analysis of each simulated dataset consumed 4.7 h of total computation on 12 concurrently executing nodes, each with eight of 16 cores (Intel Xeon E5 class processors). As expected, the success rate is reduced as the effect size (heritability) decreases and as the standard deviation of the trait increases. We also find that the sample size of 8000 has a better success rate than 2000 as expected. In general, the four-SNP target models are also harder to detect than simpler two-SNP target models. Also, detecting target four or eight-SNP models is easier when there are 100 SNPs compared to 1000 total SNPs. It is important to note the runtime for the 1000 SNP data sets averaged five hours.

More specifically, we find that Crush-MDR succeeds in identifying the correct four simulated SNP targets out of 100 or 1000 SNPs, respectively, at least 80% of the time at the lowest heritability (0.001), with a sample size of 8000, MAF 0.4, and standard deviation of the quantitative trait 0.05. These values are consistent with real GWAS data. Further, an eight SNP target model can be reliably detected under the same parameter settings when the heritability is 0.01. In both cases, four or eight SNP target models were detected out of 1000 total SNPs, a number of SNPs that is consistent with the analysis of all genes in a biochemical pathway. For comparison, Fig. 1b shows the same analysis but with a random search instead of Crush over the same number of fitness evaluations. These results confirm that Crush-MDR is able to model complex, hierarchical relationships between multiple genetic risk factors in realistic population-level data.

### Application of Crush-MDR to the genetic analysis of Alzheimer’s disease

**Fig. 2:**
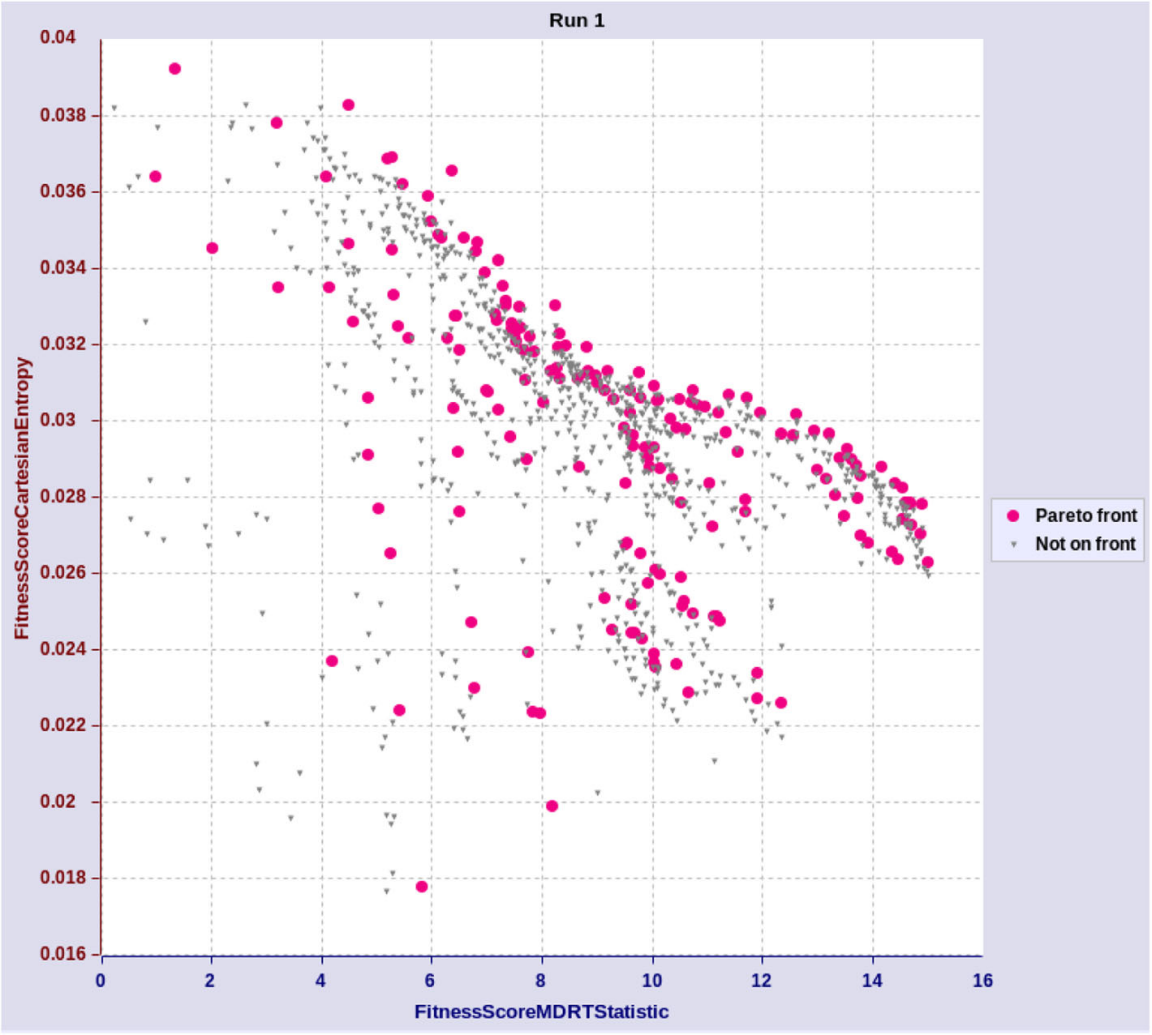
MDR t-statistic (*x-axis*) vs. Cartesian entropy (*y-axis*) results for the Crush-MDR multiobjective optimization analysis of normalized hippocampal volume in the ADNI dataset, as visualized in the Crush-MDR visualization module. Models on the Pareto front are shown as *pink points*, and all other models explored by Crush-MDR in the run are shown as *gray points*

**Fig. 3:**
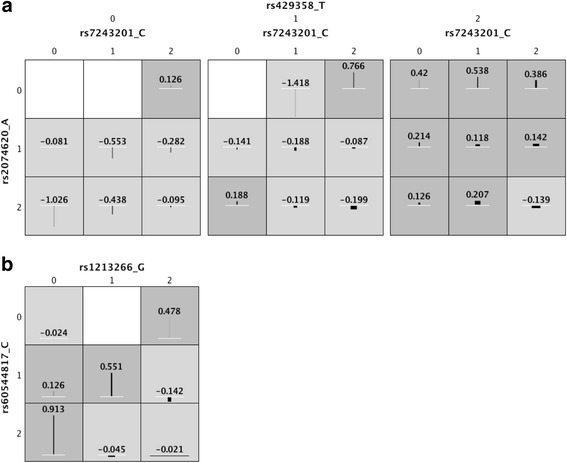
Associations between genotype and phenotype for the top-scoring (**a**) three-factor and (**b**) two-factor models on the Crush-MDR Pareto front. Each cell shows one genotype combination and the average phenotypic difference from the mean for subjects with those genotypes. Wider bars indicate a larger number of subjects with those genotypes. *Dark gray cells* indicate genotypes associated with *higher*-than-average hippocampal volume (HV). *Light gray* cells indicate *lower*-than-average HV, and *white cells* represent genotypes with no subjects. Genotypes are coded as 0/1/2 according to the number of copies of the major allele

Out of an input set containing 758 SNPs in 28 genes, Crush-MDR analysis of normalized HV in the ADNI study identified 180 models on the Pareto front (Fig. 2) containing 136 unique SNPs in 23 genes. The association between genotype and phenotype for each genotype combination in the two- and three-factor models with the highest MDR t-statistic scores are shown in Fig. 3. In the three-factor model (Fig. 3a), rs429358 is one of two main SNPs in the Apolipoprotein E (APOE) gene associated with late-onset Alzheimer’s disease (LOAD), with the C allele being pathogenic. In this interaction, the main effect of this SNP can be seen, with the zero and one genotypes having generally smaller HV, which is associated with LOAD. However, there are some genotype combinations in this interaction that do not fit the simple main effect association. For example, subjects homozygous for the non-pathogenic allele (rs429358_T_2) show *smaller* than average HV when combined with rs7243201_C_2 and rs2074620_A_2. These kinds of non-additive interactions could help explain why some subjects without high-risk alleles are affected by LOAD. In the two-factor model (Fig. 3b), rs1213266 has a weak individual effect (p = 0.00240), but this effect is clearly modulated by the genotype at rs60544817, which has no individual effect (p = 0.448). For example, the heterozygous genotype rs1213266_G_1 is protective (larger HV) when paired with rs60544817_C_1 but pathogenic (smaller HV) when paired with rs60544817_C_2.

**Fig. 4:**
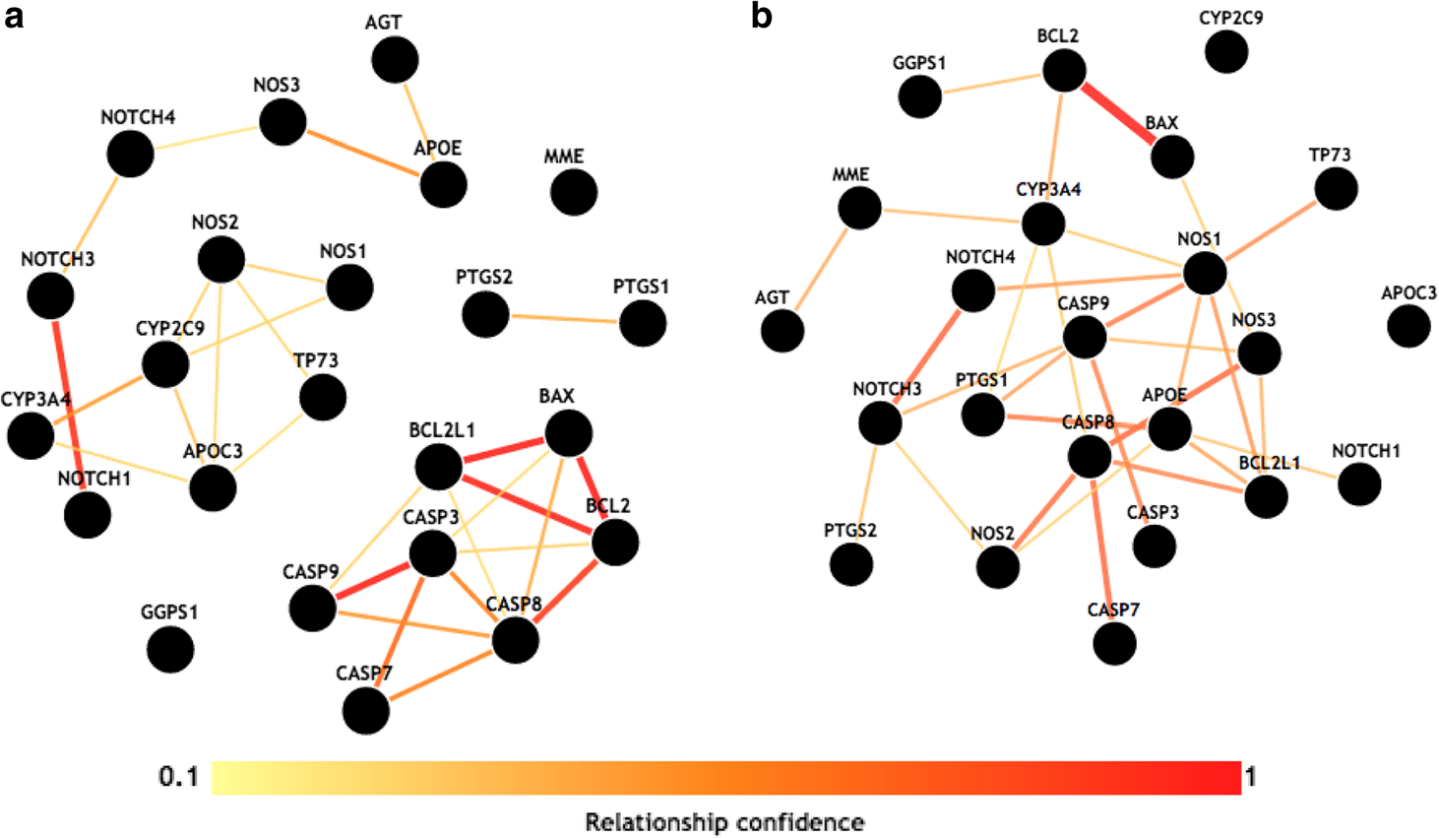
**a** IMP and **b** GIANT neuron analysis of the 19 genes found on the Pareto front of the Crush-MDR analysis of normalized hippocampal volume in the ADNI dataset. The minimum relationship confidence in each figure is 0.2

The set of genes on the Pareto front was analyzed using the Integrative Multi-Species Prediction (IMP) webserver, [49] which evaluates biological evidence for functional interactions between genes (Fig. 4a). In IMP, the strength of the functional interaction between each pair of genes is estimated using a Bayesian algorithm that considers gene co-expression across thousands of gene expression datasets in addition to factors such as protein-protein interaction, association with the same disease, being part of the same pathway, or sharing transcription factor binding sites. This gene set was also analyzed using the related Genome-scale Integrated Analysis of gene Networks in Tissues (GIANT) webserver, [50] which looks for functional interactions within a particular human tissue, the neuron in this case (Fig. 4b). Both approaches find strong evidence for biological interactions among the genes on the Pareto front, including high levels of co-expression, physical interactions, and shared transcription factor binding. Between the two analyses, all Pareto front genes have at least one biological interaction with another Pareto front gene at a confidence ≥ 0.2. These functional genomics analyses provide base level validation that Crush-MDR successfully identified a set of interacting genes with biological ties to Alzheimer’s disease in genome-wide data.

## Discussion

We have entered the golden era of bioinformatics [51] marking a shift of focus from the technology used to generate big data to the computational methods and software for making sense of it. Univariate analyses of GWAS data have yielded numerous associations of small effect accounting for 20% or less of the heritability of most common diseases. It is our working hypothesis that much of the remaining heritability is due to context-dependent effects. That is, each genetic variant will impact disease susceptibility in the context of its genomic background and local ecology defined by a history of environmental exposure. Machine learning and artificial intelligence methods will play an important role in modeling these context-dependent effects, which will sometimes present as non-additive interactions between sets of genetic variants and/or environmental factors. We have explored here the use of the MDR algorithm for the detection of gene-gene interactions using a stochastic search framework implemented in a cloud-computing environment. Both simulation and real data analyses suggest that Crush-MDR is able to identify non-additive gene-gene interactions in genome-wide data.

There are several lessons learned from this study and our previous studies mentioned above. First, it is not computationally feasible to enumerate all three-way, four-way, and higher-order genetic models in genome-wide studies. The combinatorial explosion of the model space makes this impossible given global computing resources. Quantum computing holds some promise for these high-order models. In the meantime, we recommend applying statistical, computational, or biological filters to reduce the set of genetic variants to a computationally tractable number prior to machine learning analysis [4]. This could take the form of expert knowledge derived from databases such as Gene Ontology or literature sources such as PubMed. Even then, stochastic search is necessary to explore the model space. Second, we need to move away from evaluating models based on a single criterion such as a p-value from a statistical test. Many other objectives will be equally important. Here, we used entropy-based measures of interaction information as one of three objectives for evaluating model quality. This could easily be another important criterion such as the drugability of the genes in the model. Third, machine learning is by nature computationally intensive. We implemented Crush-MDR in a cloud-computing environment, as cloud services are increasingly available and inexpensive compared to local parallel computing clusters. We expect these technologies to play an important role in the detection and characterization of gene-gene interactions using methods such as Crush-MDR.

The most important and most challenging aspect of this kind of analysis is the pre-processing of the data to reduce the search space of Crush-MDR. Here we used biological knowledge to select a subset genes and their SNPs representing an Alzheimer’s disease pathway prior to analysis. Other filtering methods such as ReliefF algorithm could be used to select a smaller subset of SNPs that are more likely to interact. ReliefF and its extensions have been shown to be effective at filtering SNPs with interaction effects [30]. We used pairwise entropy-based measures of interaction information as expert knowledge in Crush-MDR. These are relatively fast computational methods that can be used to prioritize SNPs for gene-gene interaction analysis using machine learning methods. These pairwise interaction information measures could also be used as a filter with an appropriate threshold. An overview of filter approaches is provided by Ritchie [52].

## Conclusions

The future success of this kind of analysis will depend primarily on the expert knowledge that is used to filter big data to a manageable size and the availability of inexpensive high-performance computing. It is our position that the time is now to tackle the search for combinations of genetic variants that interact to influence disease risk. Functional genomics data made available through tools such as IMP [49] and increasing availability of inexpensive cloud computing due to competition between vendors will enable methods such as Crush-MDR to be used for large-scale genetic analyses.

## References

[1] Cowper-Sal Lari R, Cole MD, Karagas MR, Lupien M, Moore JH (2011). Layers of epistasis: genome-wide regulatory networks and network approaches to genome-wide association studies. Wiley Interdiscip. Rev Syst Biol Med.

[2] Moore JH (2003). The ubiquitous nature of epistasis in determining susceptibility to common human diseases. Hum Hered.

[3] Cordell HJ (2009). Detecting gene-gene interactions that underlie human diseases. Nat Rev Genet.

[4] Moore JH, Asselbergs FW, Williams SM (2010). Bioinformatics challenges for genome-wide association studies. Bioinforma Oxf Engl.

[5] Bateson W (1907). The progress of genetics since the rediscovery of Mendel’s paper. Progress Rei Bot.

[6] Phillips PC (1998). The language of gene interaction. Genetics.

[7] Fisher RA (1918). The correlation between relatives on the supposition of Mendelian inheritance. Trans R Soc Edinb.

[8] Moore JH (2005). A global view of epistasis. Nat Genet.

[9] Moore JH, Williams SM (2005). Traversing the conceptual divide between biological and statistical epistasis: systems biology and a more modern synthesis. BioEssays News Rev Mol Cell Dev Biol.

[10] Mckinney BA, Reif DM, Ritchie MD, Moore JH (2006). Machine learning for detecting gene-gene interactions: a review. Appl Bioinformatics.

[11] Thornton-Wells TA, Moore JH, Haines JL (2004). Genetics, statistics and human disease: analytical retooling for complexity. Trends Genet.

[12] Motsinger AA, Ritchie MD, Reif DM (2007). Novel methods for detecting epistasis in pharmacogenomics studies. Pharmacogenomics.

[13] Nelson MR, Kardia SL, Ferrell RE, Sing CF (2001). A combinatorial partitioning method to identify multilocus genotypic partitions that predict quantitative trait variation. Genome Res.

[14] Culverhouse R, Klein T, Shannon W (2004). Detecting epistatic interactions contributing to quantitative traits. Genet Epidemiol.

[15] Ritchie MD, Hahn LW, Roodi N, Bailey LR, Dupont WD, Parl FF (2001). Multifactor-dimensionality reduction reveals high-order interactions among estrogen-metabolism genes in sporadic breast cancer. Am J Hum Genet.

[16] Hahn LW, Ritchie MD, Moore JH (2003). Multifactor dimensionality reduction software for detecting gene-gene and gene-environment interactions. Bioinforma Oxf Engl.

[17] Kooperberg C, Ruczinski I (2005). Identifying interacting SNPs using Monte Carlo logic regression. Genet Epidemiol.

[18] Kooperberg C, Ruczinski I, Leblanc ML, Hsu L (2001). Sequence analysis using logic regression. Genet Epidemiol.

[19] Ritchie MD, Hahn LW, Moore JH (2003). Power of multifactor dimensionality reduction for detecting gene-gene interactions in the presence of genotyping error, missing data, phenocopy, and genetic heterogeneity. Genet Epidemiol.

[20] Hahn LW, Moore JH (2004). Ideal discrimination of discrete clinical endpoints using multilocus genotypes. In Silico Biol.

[21] Moore JH, Gilbert JC, Tsai C-T, Chiang F-T, Holden T, Barney N (2006). A flexible computational framework for detecting, characterizing, and interpreting statistical patterns of epistasis in genetic studies of human disease susceptibility. J Theor Biol.

[22] Moore JH, Andrews PC (2015). Epistasis analysis using multifactor dimensionality reduction. Methods Mol Biol.

[23] Michalski RS (1983). A theory and methodology of inductive learning. Artif Intel.

[24] Hastie T, Tibshirani R, Friedman J. Elements of Statistical Learning: data mining, inference, and prediction [Internet]. Springer; 2009 [Cited 2016 Dec 12]. Available from: http://statweb.stanford.edu/~tibs/ElemStatLearn//

[25] Velez DR, White BC, Motsinger AA, Bush WS, Ritchie MD, Williams SM (2007). A balanced accuracy function for epistasis modeling in imbalanced datasets using multifactor dimensionality reduction. Genet Epidemiol.

[26] Moore JH. A user-friendly open-source MDR software package written in Java [Internet]. Available from: www.epistasis.org

[27] Sinnott-Armstrong NA, Greene CS, Cancare F, Moore JH (2009). Accelerating epistasis analysis in human genetics with consumer graphics hardware. BMC Res Notes.

[28] Greene CS, Sinnott-Armstrong NA, Himmelstein DS, Park PJ, Moore JH, Harris BT (2010). Multifactor dimensionality reduction for graphics processing units enables genome-wide testing of epistasis in sporadic ALS. Bioinformatics.

[29] Moore JH, White BW, Moore JH, Rajapakse JC, Marchiori E (2007). Tuning relieff for genome-wide genetic analysis. Evolutionary computation, machine learning and data mining, bioinformatics.

[30] Greene CS, Penrod NM, Kiralis J, Moore JH (2009). Spatially uniform relieff (SURF) for computationally-efficient filtering of gene-gene interactions. BioData Min.

[31] Pattin KA, Moore JH (2008). Exploiting the proteome to improve the genome-wide genetic analysis of epistasis in common human diseases. Hum Genet.

[32] Sullivan K, Luke S, Larock C, Cier S, Armentrout S. Opportunistic Evolution: Efficient Evolutionary Computation on Large-scale Computational Grids. Proc. 10th Annu. Conf. Companion Genet. Evol. Comput. [Internet]. New York, NY, USA: ACM; 2008 [Cited 2016 Dec 12]. p. 2227–32. Available from: http://doi.acm.org/10.1145/1388969.1389050

[33] Gui J, Moore JH, Williams SM, Andrews P, Hillege HL, van der Harst P (2013). A Simple and Computationally Efficient Approach to Multifactor Dimensionality Reduction Analysis of Gene-Gene Interactions for Quantitative Traits. Plos One.

[34] De R, Verma SS, Holzinger E, Hall M, Burt A, Carrell DS (2016). Identifying gene-gene interactions that are highly associated with four quantitative lipid traits across multiple cohorts. Hum Genet.

[35] Deb K, Pratap A, Agarwal S, Meyarivan T (2002). A Fast and Elitist Multiobjective Genetic Algorithm: NSGA-II. Trans Evol Comp.

[36] Moore JH, Hu T (2015). Epistasis analysis using information theory. Methods Mol Biol.

[37] Hu T, Sinnott-Armstrong NA, Kiralis JW, Andrew AS, Karagas MR, Moore JH (2011). Characterizing genetic interactions in human disease association studies using statistical epistasis networks. BMC Bioinformatics.

[38] Coello Coello CA (2002). Theoretical and numerical constraint-handling techniques used with evolutionary algorithms: a survey of the state of the art. Comput Methods Appl Mech Eng.

[39] Smits GF, Kotanchek M. Pareto-Front Exploitation in Symbolic Regression. In: O’Reilly U-M, Yu T, Riolo R, Worzel B, editors. Genet. Program. Theory Pract. II [Internet]. Springer US; 2005 [cited 2016 Dec 12]. p. 283–99. Available from: http://link.springer.com/chapter/10.1007/0-387-23254-0_17

[40] Moore JH, Hill DP, Sulovari A, Kidd LC. Genetic Analysis of Prostate Cancer Using Computational Evolution, Pareto-Optimization and Post-processing. In: Riolo R, Vladislavleva E, Ritchie MD, Moore JH, editors. Genet. Program. Theory Pract. X [Internet]. Springer New York; 2013 [cited 2016 Dec 12]. p. 87–101. Available from: http://link.springer.com/chapter/10.1007/978-1-4614-6846-2_7

[41] Moore JH, Greene CS, Hill DP. Identification of Novel Genetic Models of Glaucoma Using the “EMERGENT” Genetic Programming-Based Artificial Intelligence System. In: Riolo R, Worzel WP, Kotanchek M, editors. Genet. Program. Theory Pract. XII [Internet]. Springer International Publishing; 2015 [cited 2016 Dec 12]. p. 17–35. Available from: http://link.springer.com/chapter/10.1007/978-3-319-16030-6_2

[42] Mell P, Grance. The NIST definition of cloud computing [Recommendations of the National Institute of Standards and Technology-Special Publication 800–145] [Internet]. Washington DC: NIST; 2011 [cited 2016 Dec 12]. Available from: http://nvlpubs.nist.gov/nistpubs/Legacy/SP/nistspecialpublication800-145.pdf

[43] Parabon Computation Inc. A parallel cloud computing infrastructure [Internet]. Available from: www.parabon.com

[44] Urbanowicz RJ, Kiralis J, Fisher JM, Moore JH (2012). Predicting the difficulty of pure, strict, epistatic models: metrics for simulated model selection. BioData Min.

[45] Urbanowicz RJ, Kiralis J, Sinnott-Armstrong NA, Heberling T, Fisher JM, Moore JH (2012). GAMETES: a fast, direct algorithm for generating pure, strict, epistatic models with random architectures. BioData Min.

[46] Mueller SG, Weiner MW, Thal LJ, Petersen RC, Jack C, Jagust W (2005). The Alzheimer’s disease neuroimaging initiative. Neuroimaging Clin N Am.

[47] Purcell S, Neale B, Todd-Brown K, Thomas L, Ferreira MAR, Bender D (2007). PLINK: a tool set for whole-genome association and population-based linkage analyses. Am J Hum Genet.

[48] Bush WS, Dudek SM, Ritchie MD. Biofilter: a knowledge-integration system for the multi-locus analysis of genome-wide association studies.Pac Symp Biocomput. 2009;368–79. PMC2859610 19209715

[49] Wong AK, Krishnan A, Yao V, Tadych A, Troyanskaya OG (2015). IMP 2.0: a multi-species functional genomics portal for integration, visualization and prediction of protein functions and networks. Nucleic Acids Res.

[50] Greene CS, Krishnan A, Wong AK, Ricciotti E, Zelaya RA, Himmelstein DS (2015). Understanding multicellular function and disease with human tissue-specific networks. Nat Genet.

[51] Moore JH, Holmes JH (2016). The golden era of biomedical informatics has begun. BioData Min.

[52] Ritchie MD (2011). Using biological knowledge to uncover the mystery in the search for epistasis in genome-wide association studies. Ann Hum Genet.

